# Enhancing milk quality and modulating rectal microbiota of dairy goats in starch-rich diet: the role of bile acid supplementation

**DOI:** 10.1186/s40104-023-00957-7

**Published:** 2024-01-22

**Authors:** Qingyan Yin, Junjian Yu, Jiaxiao Li, Tianci Zhang, Tianyu Wang, Yufei Zhu, Jun Zhang, Junhu Yao

**Affiliations:** 1 College of Animal Science and Technology, Northwest A&F University, Yangling, 712100 Shaanxi P.R. China; 2 Key Laboratory of Livestock Biology, Northwest A&F University, Yangling, 712100 Shaanxi P.R. China; 3 DAYU Bioengineering (Xi’an) Industrial Development Research Institute, Xi’an, 710000 Shaanxi P.R. China

**Keywords:** Bile acids, Dairy goats, Lipid metabolism, Gut microbiota

## Abstract

**Background:**

Diets rich in starch have been shown to increase a risk of reducing milk fat content in dairy goats. While bile acids (BAs) have been used as a lipid emulsifier in monogastric and aquatic animals, their effect on ruminants is not well understood. This study aimed to investigate the impact of BAs supplementation on various aspects of dairy goat physiology, including milk composition, rumen fermentation, gut microbiota, and BA metabolism.

**Results:**

We randomly divided eighteen healthy primiparity lactating dairy goats (days in milk = 100 ± 6 d) into two groups and supplemented them with 0 or 4 g/d of BAs undergoing 5 weeks of feeding on a starch-rich diet. The results showed that BAs supplementation positively influenced milk yield and improved the quality of fatty acids in goat milk. BAs supplementation led to a reduction in saturated fatty acids (C16:0) and an increase in monounsaturated fatty acids (*cis*-9 C18:1), resulting in a healthier milk fatty acid profile. We observed a significant increase in plasma total bile acid concentration while the proportion of rumen short-chain fatty acids was not affected. Furthermore, BAs supplementation induced significant changes in the composition of the gut microbiota, favoring the enrichment of specific bacterial groups and altering the balance of microbial populations. Correlation analysis revealed associations between specific bacterial groups (*Bacillus* and *Christensenellaceae R-7 group*) and BA types, suggesting a role for the gut microbiota in BA metabolism. Functional prediction analysis revealed notable changes in pathways associated with lipid metabolism, suggesting that BAs supplementation has the potential to modulate lipid-related processes.

**Conclusion:**

These findings highlight the potential benefits of BAs supplementation in enhancing milk production, improving milk quality, and influencing metabolic pathways in dairy goats. Further research is warranted to elucidate the underlying mechanisms and explore the broader implications of these findings.

**Supplementary Information:**

The online version contains supplementary material available at 10.1186/s40104-023-00957-7.

## Background

Milk fat is an important source of nutrition in dairy products like butter and cream, which are commonly consumed in daily life [1]. Comprised of a complex mixture of triglycerides, milk fat provides essential fatty acids (FAs) that play crucial roles in human nutrition [2]. However, the nutritional value of milk is related closely with its FA composition, as different types of milk exhibit distinct characteristics. Goat milk, in particular, has garnered attention for its nutritional similarities to human milk [3]. We have chosen dairy goats as our experimental model due to the unique nutritional characteristics of goat milk and its resemblance to human milk composition. Mounting evidence suggests that goat milk may serve as a superior source of dairy products for regular consumption compared to cow milk [4]. Mammary synthesis of milk fat remains an active area of research, with significant progress made in understanding the regulation of lipid synthesis through various factors such as diet nutrition or additives. Milk FAs are synthesized in the mammary gland and can also be directly absorbed from the blood. Mammary milk fat is synthesized from de novo synthesized FAs (< 16C) and the direct absorption of FAs (> 16C) from the bloodstream [5]. Dietary fats undergo ruminal biohydrogenation [6], and are subsequently absorbed into the mesenteric vein, where they bind with bile acids (BAs) before entering the mammary gland [7]. However, the role of BAs in milk fat metabolism, particularly in ruminants like dairy goats, is not well understood. BAs are known to play a crucial role in lipid metabolism, acting as emulsifiers and enhancers of lipid digestion and absorption in the small intestine.

The digestion and absorption of lipids, including FAs, primarily occur in the small intestine. Upon the presence of lipids, cholecystokinin is secreted, stimulating the excretion of bile and lipase. Lipase breaks down triglycerides into glycerol and free FAs, facilitating their absorption. BAs play a critical role in this process by acting as emulsifiers, enhancing the interaction of lipase with lipids and increasing lipase activity. BAs augment the interfacial area for lipase, resulting in efficient lipid digestion and absorption in the small intestine [8]. Moreover, as signaling molecules, BAs play a crucial role in regulating lipid metabolism [9], glucose metabolism [10], and other energy metabolism pathways [11]. BAs have been used as a lipid emulsifier in monogastric and aquatic animals, their effect on ruminants is not well understood. The primary bile acid (PBA) synthesized in the liver undergo additional microbial modifications in the intestine, including deconjugation, dehydroxylation, oxidation, and epimerization. These processes lead to the formation of various secondary bile acid (SBA) [12]. Although the majority of BAs are reabsorbed in the terminal part of the small intestine (ileum) and transported back to the liver through the hepatic portal vein, a portion of BAs escapes reabsorption and reaches the hindgut. Even in this lower intestinal segment, BAs continue to exert significant effects on various physiological processes [13].

SBAs are recognized as metabolites of microorganisms and are involved in a complex interactive relationship with the gut microbiota [14]. The intestinal microorganisms further metabolize PBA, transforming them into SBA. These modified BAs have the ability to modulate the composition of the microbiota, thereby influencing the metabolism and immune function of the host organism [15]. The interplay between BAs, gut microbiota, and lipid metabolism represents a fascinating area of research with implications for understanding goat milk FA synthesis. In recent years, the role of BAs in modulating the gut microbiota and its impact on host metabolism has gained significant attention [16]. Understanding the complex interplay between BAs, the gut microbiota, and host metabolism is crucial for unraveling the potential applications of BAs in ruminants. The regulation of BAs and their interactions with the microbiota hold great potential for managing lipid metabolism in ruminants.

Given the crucial role of milk fat in human nutrition and the increasing recognition of the impact of BAs on various physiological processes. In our study, we aim to explore the effects of supplementing BAs in the diet on the composition of the rectum microbiota and BAs and their potential role in milk fat synthesis. By exploring the interactions between BAs, milk fat, and the rectum microbiota, we aim to shed light on the complex interplay between diet, microbial metabolism, and host health. This knowledge will contribute to a better understanding of the interactions between diet, gut microbiota, and milk fat metabolism, and may have implications for optimizing milk fat production and quality in dairy farming practices.

## Materials and methods

### Experimental design and sample collections

Eighteen healthy primiparous dairy goats were randomly allocated into two groups according to their day in milk (100 ± 6.0 d), body weight (45 ± 3.7 kg), and milk yield (1.6 ± 0.31 kg) (mean ± standard error). The goats were individually administered either 0 or 4 g/d of BAs and twice daily (morning and evening) using equivalent doses. The experiment period was 35 d (28 d trail period and 7 d sampling period), and we selected a purely BAs mix (from a company) for dairy goats, consisting of hyocholic acid (HCA), hyodeoxycholic acid (HDCA), and chenodeoxycholic acid (CDCA) with a minimum concentration of 95%. Throughout the experiment, the goats were offered ad libitum diet and water. The dairy goats are housed in group pens within areas measuring approximately 15 m × 3 m for feeding and resting, alongside expansive exercise spaces spanning 15 m × 20 m. The total mixed ration (TMR) as presented in Table S1, was supplied twice a day at 0530 and 1730, and the goats were subjected to twice-daily milking sessions using an electric milking machine. At the sampling period, the dairy goats were individually housed in feeding areas measuring 1.5 m × 2 m for 3 d, where ample diet and water were provided. The milk yield of goats was assessed for three consecutive days, and samples (rumen fluid, blood, milk and hindgut content) were collected.

### Diet chemical analysis

Once per week, dietary samples were collected and subsequently pulverized using a Wiley mill with a 2-mm screen, followed by a 1-mm mesh screen after 48 h of drying at 55 °C in a forced air oven to be dried. According to AOAC International [17], the samples were examined for dry matter for 8 h at 105 °C, the crude protein was determined using method #988.05, while the neutral detergent fiber, acid detergent fiber, and starch (Megazyme, Bray, Ireland) were analyzed following the protocol of Van Soest et al. [18].

### Blood metabolites analysis

The blood samples were collected from jugular vein of the dairy goats prior to morning feeding. The collected blood was then transferred to tubes containing EDTA-K_2_ and immediately placed on the ice during transportation to the laboratory for processing. Then, the collected blood samples were subjected to centrifugation at 1,000 × *g* for 20 min at 4 °C, after centrifugation, the plasma was carefully transferred into polystyrene tubes using a plastic transfer pipette and stored at −80 °C until analysis. Plasma samples were analyzed using an automated blood analyzer (Celercare V5, MNCHIP, China), to measure total of 13 blood indicators, including total protein, albumin, globulin, albumin/globulin, alanine aminotransferase, aspartate aminotransferase, aspartate aminotransferase/alanine aminotransferase, γ-glutamyl transferase, alkaline phosphatase, total bile acid (TBA), triglycerides, cholesterol (CHOL), glucose.

### Rumen fermentation parameters

Ruminal fluid samples were collected during the sampling period, 2 h after the morning feeding, using an oral rumen tube and a hand vacuum pump. To reduce saliva contamination, 50 mL of ruminal fluid was removed prior to sample collection. The collected ruminal fluid was then prepared for measuring short-chain fatty acids (SCFAs), following the protocol outlined by Ren et al. [19]. Specifically, the ruminal fluid was subjected to centrifugation at 13,500 × *g* for 10 min at 4 °C, and 1 mL of the supernatant was mixed with 200 μL of metaphosphoric acid and stored at −80 °C until further analysis. The concentration of SCFA was measured by gas chromatography (GC) using crotonic acid as an internal standard. The cleaned-up samples were then injected into the GC equipped with a fused silica capillary column (DB-FFAP, 30 m × 0.32 mm × 0.25 μm), an iron trap mass detector, and a thermal conductivity detector.

### Milk fatty acid composition analysis

Daily milk samples were obtained by pooling morning and evening milk yields into 5-mL tubes and subsequently frozen at −80 °C until analysis. The FA composition of the milk samples was analyzed using GC as described by Zheng et al. [20]. Briefly, the milk samples were methylated with 4 mL of 0.5 mol/L NaOH/methanol at 50 °C for 15 min, followed by treatment with 4 mL of 5% HCl/methanol at 50 °C for 1 h. After extraction with 2 mL of heptane, the samples were put into an Agilent 7890B GC (Agilent, Santa Clara, CA, USA) equipped with an iron trap mass detector and a fused silica capillary column (HP-88, 100 m × 0.25 mm × 0.2 μm). An internal standard of nonadecanoic acid methyl ester (Sigma, N5377) was used, and an external standard mixture of Supelco 37 component FAME mix (Sigma, CRM47885), ME61, ME93, BR2, and BR3 (Larodan Fine Chemicals AB, Malmo, Sweden) was employed.

### DNA extraction and 16S rRNA gene sequencing

DNA of hindgut content samples from 18 dairy goats was extracted using the E.Z.N.A.®Stool DNA kit (Omega Bio-Tek, Norcross, GA, USA) according to the manufacturer’s protocol. The DNA concentration was measured with a Nanodrop-2000 (Thermo Fisher Scientific, Wilmington, DE, USA) and the quality was assessed using 1% agarose gel electrophoresis. Bacterial 16S rRNA gene fragments (V3–V4) in the extracted DNA were amplified using the forward primers 338F (5′-ACT CCT ACG GGA GGC AGC AG-3′) and the reverse primer 806R (5′-GGA CTA CHVGGG TWT CTAAT-3′) [21]. PCR products were visualized on 2% agarose gels and purified using the QIAquick gel extraction kit (Qiagen, Dusseldorf, Germany). All amplicons were sequenced using the paired-end (PE300) method on a MiSeq platform (Illumina, San Diego, USA) following the standard protocols.

### Illumina sequencing data analysis

The raw sequences were merged with FLASH (v1.2.11) [22] and the quality filtered with fastp (0.19.6) [23]. Sequences were imported into QIIME2 v2021.8 for demultiplexing and the construction of an amplicon sequence variant (ASV) table using DADA2 [24]. Bacterial 16S ASVs were assigned a taxonomy using the SILVA database (version 138) as the reference, singletons were removed and a table of ASV counts per sample was generated. For the downstream analysis, we included microbial taxa with a relative abundance greater than 0.01% in more than 50% of the samples. Meanwhile, we performed rarefaction based on the smallest number of sequences in the samples to minimize the impact on subsequent analysis. Furthermore, phylogenetic investigation of communities by reconstruction of unobserved states 2 (PICRUSt2) analysis (https://github.com/picrust/picrust2) [25] was used to predict the metagenome based on the ASV table, and then the metagenome functions were predicted, and the data were of Kyoto Encyclopedia of Genes and Genomes (KEGG) database pathways. Alpha diversity indices including the richness estimate and Shannon diversity index were calculated using QIIME 2 at the ASV level. The principal component analysis (PCA) statistical significance was determined using an analysis of similarities (Adonis) with 999 permutations at the genus level based on Bray–Curtis. To identify the significantly abundant bacterial taxa (from phylum to genus level) among the different groups, we conducted linear discriminant analysis (LDA) effect size (LEfSe) analysis [26] (http://huttenhower.sph.harvard.edu/LEfSe). This analysis allowed us to determine the taxa (phylum to genera) that showed significant differences in abundance between the groups, with an LDA score greater than 3 and a significance level of* P* < 0.05.

### BA analysis

The hindgut content samples (20 mg) were first ground using a ball mill. Subsequently, 10 μL of an internal standard mixture working solution (1 μg/mL) and 200 μL of methanol/acetonitrile (v/v = 2:8) were added for homogenization. After homogenization, the samples were shaken at 2,500 r/min for 10 min, and then the samples were kept at −20 °C for 10 min to precipitate protein, followed by centrifugation for 10 min at 12,000 r/min and 4 °C. The resulting supernatant was transferred to clean plastic microtubes and concentrated using a concentrator (CentriVap, LABCONCO, USA). Upon completion of the concentration step, the samples were reconstituted with 100 μL of 50% methanol–water solution for further LC–MS/MS applied biosystems 6500 triple quadrupole (QTRAP 6500 + , SCIEX, USA) analysis. The HPLC column used was Waters ACQUITY UPLC HSS T3 C18 (100 mm × 2.1 mm i.d., 1.8 µm) [26]. After unblinding and releasing the data, the metabolite profiles underwent quality control checks and were preprocessed to ensure data quality and robustness of subsequent analyses. The preprocessing steps included adjusting for batch effects, imputing missing values, and performing log-transformation.

### Statistical analysis

We analyzed the data using SPSS 26.0. Prior to conducting the analyses, the normality of the studentized residuals of all variables was evaluated using the Shapiro–Wilk test, and the homogeneity of variances was checked. Results indicated that all variables were normally distributed and homogeneous, meeting the necessary assumptions for the planned statistical analyses. A student’s *t*-test was performed to compare the two groups. The goats were considered as experimental units, while the experimental treatments were used as fixed effects. Blood metabolites, ruminal SCFAs, milk FAs and hindgut BAs were considered as dependent variables. The experimental design was determined to be statistically valid, with the 9 replicates meeting the statistical requirements for the observed changes in milk fat.

Results were expressed as least squares means and standard errors of the mean. A probability value of *P* < 0.05 was statistically significant, and tendencies were described when 0.05 ≤ *P* < 0.10.

## Results

### BAs supplementation increased milk yield and quality in dairy goats fed a starch-rich diet

**Fig. 1:**
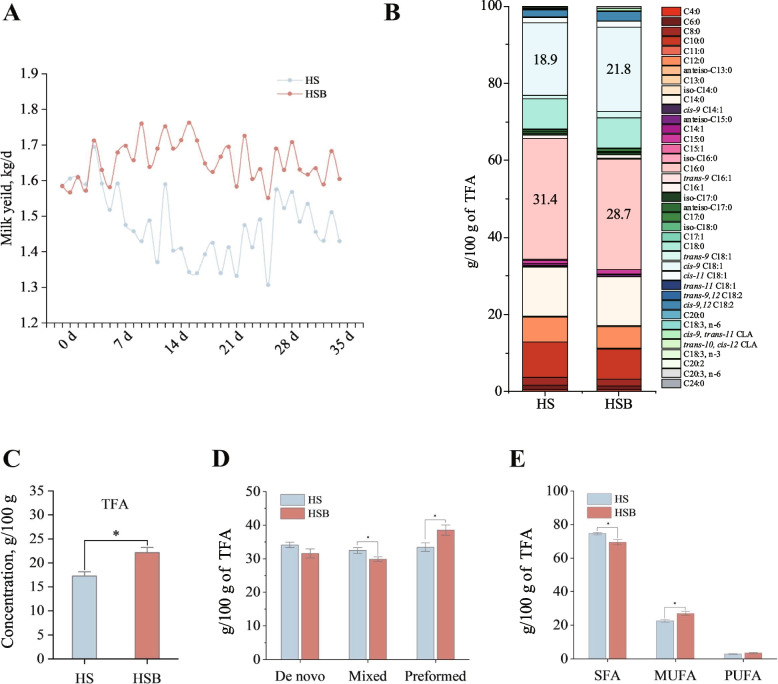
The effect of supplement bile acids on milk yield and fatty acid composition of dairy goats fed starch-rich diets (*n* = 9). **A** Average daily milk yield during the test period. **B** FA composition** C** Total fatty acid **D** Different source of milk FAs **E** Milk FA classification. HS means fed starch-rich diet, HSB means fed starch-rich diet supply 4 g/d bile acids for dairy goats every day. De novo (FAs < 16 C) originates from de novo synthesis in the mammary gland, preformed (FAs > 16 C) originate from plasma, and mixed (FAs = 16 C) originates from both sources. *FAs* Fatty acids, *TFA* Total fatty acid, *SFA* Saturated fatty acids, *MUFA* Monounsaturated fatty acids, *PUFA* Polyunsaturated fatty acids. ^*^P < 0.05

BAs supplementation had a positive effect on milk yield (Fig. 1A) and milk total fatty acid (Fig. 1C). Furthermore, we investigated the impact of BAs supplementation on the FA composition of milk in dairy goats fed a starch-rich diet (Fig. 1B). The FA composition analysis indicated that C16:0 and *cis*-9 C18:1 were the predominant FAs in goat milk. Notably, the group receiving BAs supplementation exhibited a decrease (*P* = 0.02) in the proportion of C16:0 and an increase (*P* = 0.02) in the proportion of *cis*-9 C18:1. Our results showed that BAs supplementation resulted in an increase (*P* < 0.01) in the proportion of preformed FAs, and a decrease (*P* = 0.03) in the proportion of mixed FAs, while the proportion of de novo FAs is similar between two groups (Fig. 1D). We found a decrease (*P* < 0.01) proportion of saturated fatty acids, whereas an increased (*P* < 0.01) proportion of monounsaturated fatty acids in the BAs supplementation group. However, the proportion of polyunsaturated fatty acids remained similar in both groups (Fig. 1E).

BAs supplementation resulted in a lower proportion of C10:0 (*P* = 0.02). Additionally, BAs supplementation was associated with increased proportions of C17:1 (*P* = 0.05) and C18:3, n-3 (*P* = 0.05). However, no significant differences were observed between the different treatments for other FAs (Table S2). Overall, our results suggested that the changes in the FA composition of milk were most pronounced in preformed FAs.

### Effect of BAs supplementation on ruminal SCFAs and blood metabolites in dairy goats fed starch-rich diet

**Fig. 2:**
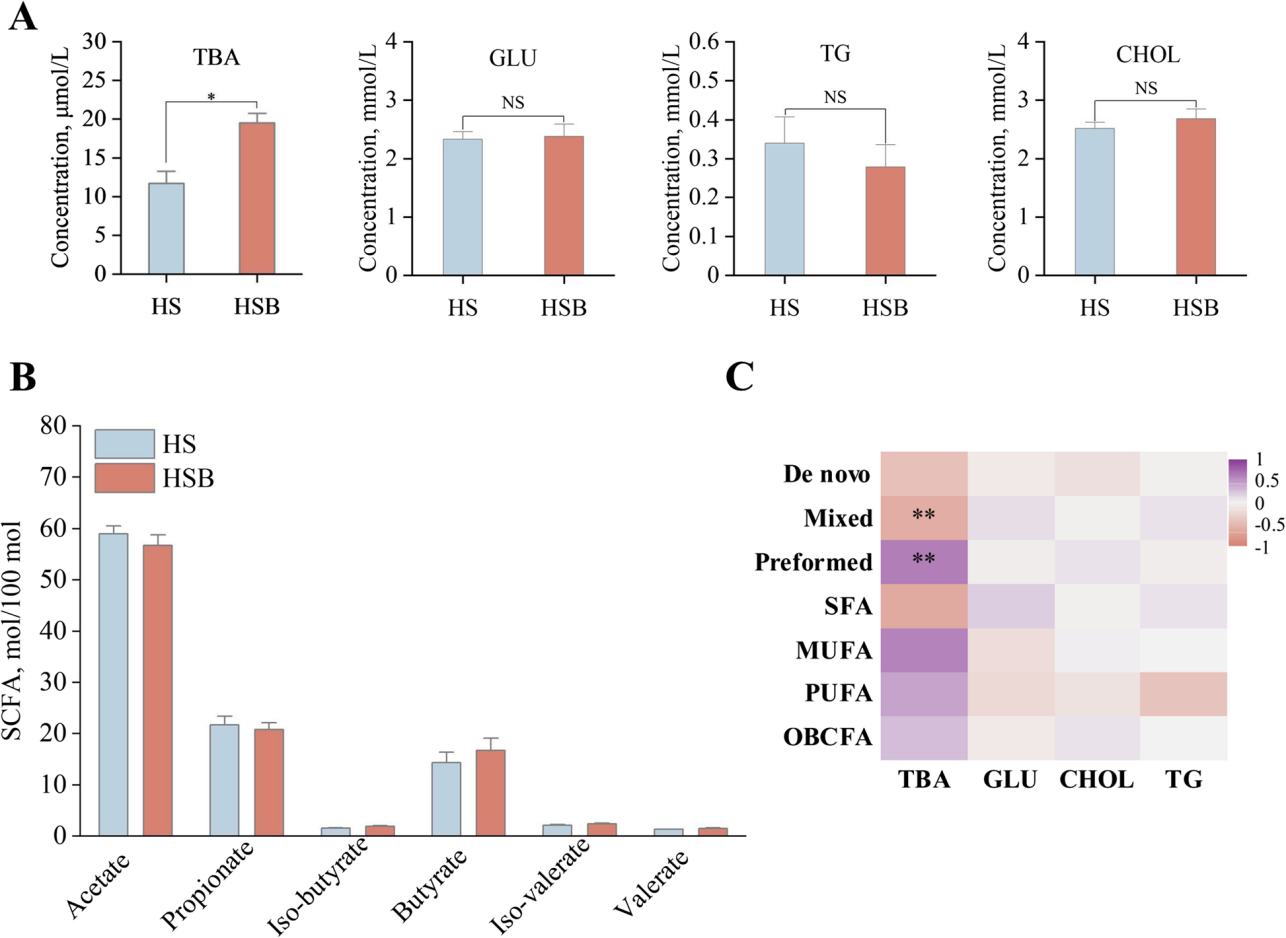
The effect of supplement bile acids on blood metabolites and rumen fermentation parameters of dairy goats fed starch-rich diets (*n* = 9). **A** Blood metabolite. **B** Rumen fermentation parameters. **C** Correlations between the milk FAs with blood metabolites. HS means fed starch-rich diet, HSB means fed starch-rich diet supply 4 g/d bile acids for dairy goats every day. *TBA* Total bile acid, *TG* Triglycerides, *CHOL* Cholesterol, *GLU* Glucose, *SFA* Saturated fatty acids, *MUFA* Monounsaturated fatty acids, *PUFA* Polyunsaturated fatty acids. De novo, preformed, and mixed De novo (FAs < 16 C) originates from de novo synthesis in the mammary gland, preformed (FAs > 16 C) originates from plasma, and mixed (FAs = 16 C) originate from both sources. ^*^P < 0.05, ^**^P < 0.01

In addition, we also investigated the effect of BAs supplementation on rumen fermentation parameters (Fig. 2A) and plasma metabolites. Our findings revealed that BAs supplementation did not significantly affect the ratio of rumen SCFAs. However, it did have a positive effect on the production of plasma TBA (*P* < 0.01) and no effect on other plasma metabolites between treatments (Fig. 2B, Table S3).

The milk FA proportion was investigated using a Spearman correlation (|*r*|> 0.6, *P* < 0.05) analysis to understand the relationship between milk FA proportion and plasma metabolites. There was a positive correlation observed between TBA and the proportion of preformed FAs, whereas a negative correlation was found between TBA and the proportion of mixed FAs (Fig. 2C). Furthermore, the proportion of *cis*-9, C18:1 was positively correlated with TBA, whereas the proportion of C10:0 and C16:0 was negatively correlated with TBA. In addition, the proportion of iso-C16:0 was found to be positively correlated with CHOL and yet C15:1 was negatively correlated with CHOL (Fig. S1A). Meanwhile, the milk FA proportion was investigated using a Spearman correlation analysis to understand the relationship between milk FA proportion and rumen fermentation parameters (Fig. S1B). The proportion of C17:1 was found to have a positive correlation with iso-butyrate, while no significant correlations were observed between other SCFAs and FAs (|*r*|> 0.6, *P* < 0.05).

### Differences in hindgut content BA composition in dairy goats fed a starch-rich diet with BAs supplementation

**Fig. 3:**
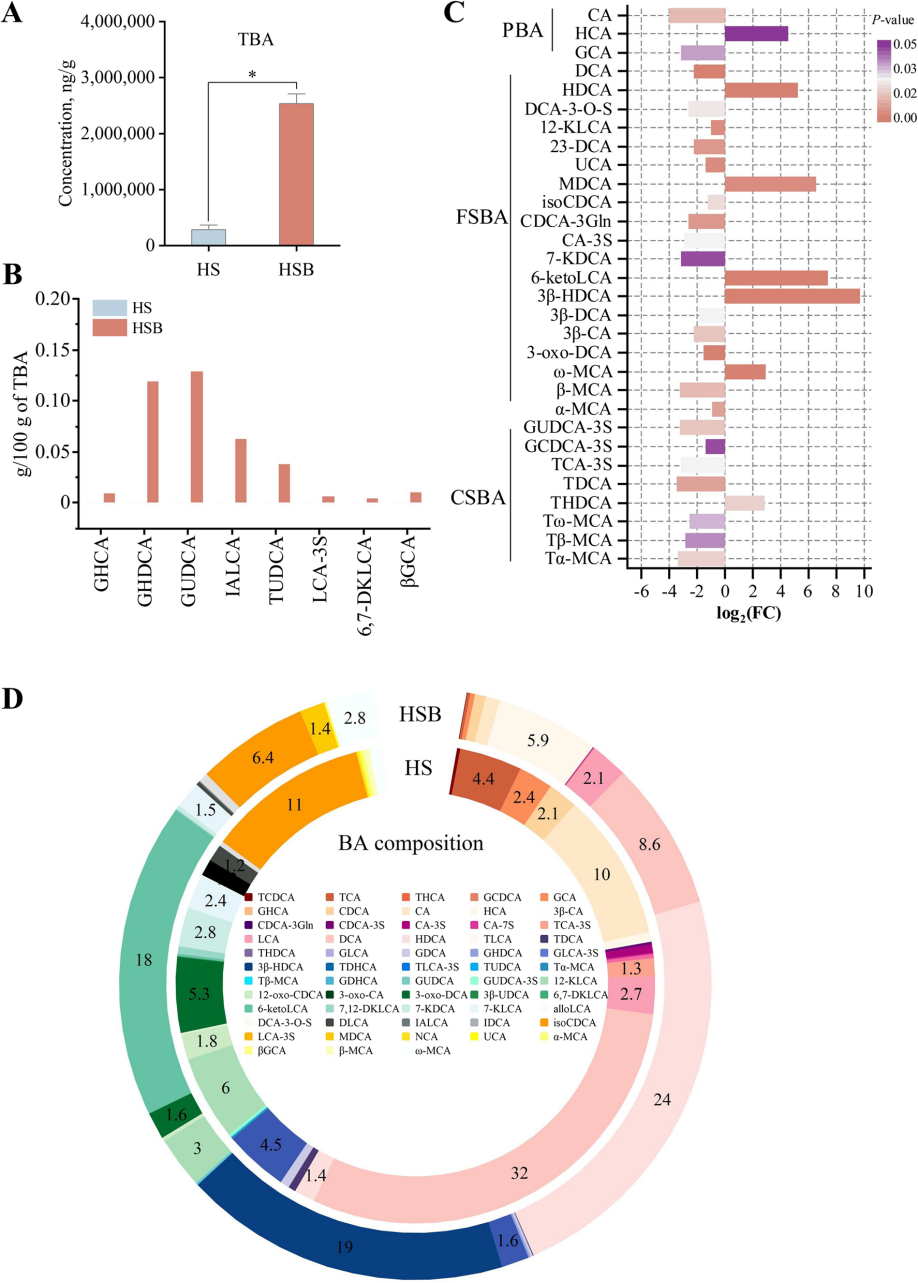
Hindgut bile acid (BA) profiles between HS and HSB group (*n* = 9).** A** The total bile acid concentration in the hindgut of HS and HSB group.** B** Bile acids unique to the HSB group. HS means fed a starch-rich diet. **C** Significantly different relative abundance of bile acids in the hindgut of HS and HAB group. **D** Relative abundance of bile acids in the hindgut of HS and HSB group. HS means fed starch-rich diet. HSB means fed a starch-rich diet supply of 4 g/d bile acids for dairy goats every day. The differences in data in (**A**) and (**B**) were tested by Wilcon rank-sum. The bars represent mean ± SEM. ^*^P < 0.05. *TBA* Total bile acids, *PBA* Primary bile acid, *FSBA* Free secondary bile acid, *CSBA* Conjugated secondary bile acid, *12-KLCA* 12-Ketolithocholic acid, *12-oxo-CDCA* 12-Oxochenodeoxycholic acid, *3-oxo-CA* 3-Oxocholic acid, *3-oxo-DCA* 3-Oxodeoxycholic acid, *3β-CA* 3β-Cholic acid, *3β-UDCA* 3β-Ursodeoxycholic acid, *6,7-DKLCA* 6,7-Diketolithocholic acid, *6-ketoLCA* 5-β-Cholanic acid-3α-ol-6-one, *7,12-DKLCA* 7,12-Diketolithocholic acid, *7-KDCA* 7-Ketodeoxycholic acid, *7-KLCA* 7-Ketolithocholic acid, *CA* Cholic acid, *CA-3S* Cholic acid 3-sulfate sodium salt, *CA-7S* Cholic acid 7-sulfate, *CDCA* Chenodeoxycholic acid, *CDCA-3Gln* Chenodeoxycholic acid-3-β-D-glucuronide, *CDCA-3S* Chenodeoxycholic acid 3-sulfate disodium salt, *DCA* Deoxycholic acid, *DCA-3-O-S* Deoxycholic acid 3-O-sulfate disodium salt, *DLCA* Dehydrolithocholic acid, *GCA* Glycocholic acid, *GCDCA* Glycochenodeoxycholic acid, *GCDCA-3S* Glycochenodeoxycholic acid 3-sulfate disodium salt, *GDCA* Glycodeoxycholic acid, *GHCA* Glycohyocholic acid, *GHDCA* Glycohyodeoxycholic acid, *GLCA* Glycolithocholic acid, *GUDCA* Glycoursodeoxycholic acid, *GUDCA-3S* Glycoursodeoxycholic acid 3-sulfate sodium, *HCA* Hyocholic acid, *HDCA* Hyodeoxycholic acid, *IALCA* Isoallolithocholic acid, *ILCA* Isolithocholic acid, *IsoCDCA* Isochenodeoxycholic acid, *LCA* Lithocholic acid, *LCA-3S* Lithocholic acid-3-sulfate, *MDCA* Murideoxycholic acid, *NCA* Norcholic acid, *TCA* Taurocholic acid, *TCA-3S* Taurocholic acid 3-sulfate sodium salt, *TCDCA* Taurochenodeoxycholic acid, *TDCA* Taurodeoxycholic acid, *THDCA* Taurohyodeoxycholic acid, *TLCA* Taurolithocholic acid, *TUDCA* Tauroursodeoxycholic acid, *Tα-MCA* Tauro-α-muricholic acid, *Tβ-MCA* Tauro-β-muricholic acid, *UCA* Ursocholic acid, *α-MCA* α-muricholic acid, *βGCA* 3β-Glycocholic acid, *β-MCA* β-Muricholic acid, *ω-MCA* ω-Muricholic acid

The Wilcon rank-sum was used to identify differentially proportioned BAs between the HS and HSB groups. The hindgut TBA concentration was higher in the HSB group compared to the HS group (Fig. 3A,  *P* < 0.01). In the HSB group, eight types of BAs include glycohyocholic acid (GHCA), glycohyodeoxycholic acid (GHDCA), glycohyodeoxycholic acid (GUDCA), isoallolithocholic acid (IALCA), tauroursodeoxycholic acid (TUDCA), lithocholic acid-3-sulfate (LCA-3S), 6,7-diketolithocholic acid (6,7-DKLCA) and 3β-glycocholic acid (βGCA) were identified, which were not found in the HS group (Fig. 3B).

Three significantly different PBAs were observed between the two groups. The relative proportion of HCA (*P* < 0.01) was higher in the HSB group, whereas the relative proportion of cholic acid (CA) (*P* < 0.01) and glycocholic acid (GCA) (*P* = 0.02) was lower in the HSB group. Regarding the SBA, 26 kinds of SBAs were identified, including 8 kinds of free secondary bile acid (FSBA) and 8 kinds of conjugated secondary bile acid (CSBA). Specifically, HDCA (*P* < 0.01), murideoxycholic acid (MDCA) (*P* < 0.01), 3β-hyodeoxycholic acid (3β-HDCA) (*P* < 0.01), 5β-cholanic acid-3α-ol-6-one (6-ketoLCA) (*P* < 0.01), ω-muricholic acid (ω-MCA) (*P* < 0.01), and taurohyodeoxycholic acid (THDCA) (*P* = 0.02) had a higher proportion in the HSB group, while other SBAs had a higher proportion in the HS group (Fig. 3C; Table S4). Among them, 3β-HDCA showed the greatest fold change (Fig. 3C, log_2_FC = 9.68) and glyco-PBA (GPBA) is showed the greatest fold change according BAs classification (Fig. S2, log_2_FC = −3.08). The analysis of BA composition revealed that in the HSB group, HDCA accounted for 24.1%, 3β-HDCA for 18.8%, and 6-ketoLCA for 18.1% of the total BA in the goat hindgut content (Fig. 3D, Table S4). In contrast, in the HS group, deoxycholic acid (DCA) accounted for 31.5% and isochenodeoxycholic acid (iso-CDCA) accounted for 11.5% of the total BA in goat hindgut content (Fig. 3D, Table S4).

### Differences in rectum microbiota structure and composition in dairy goats fed a starch-rich diet with BAs supplementation

**Fig. 4:**
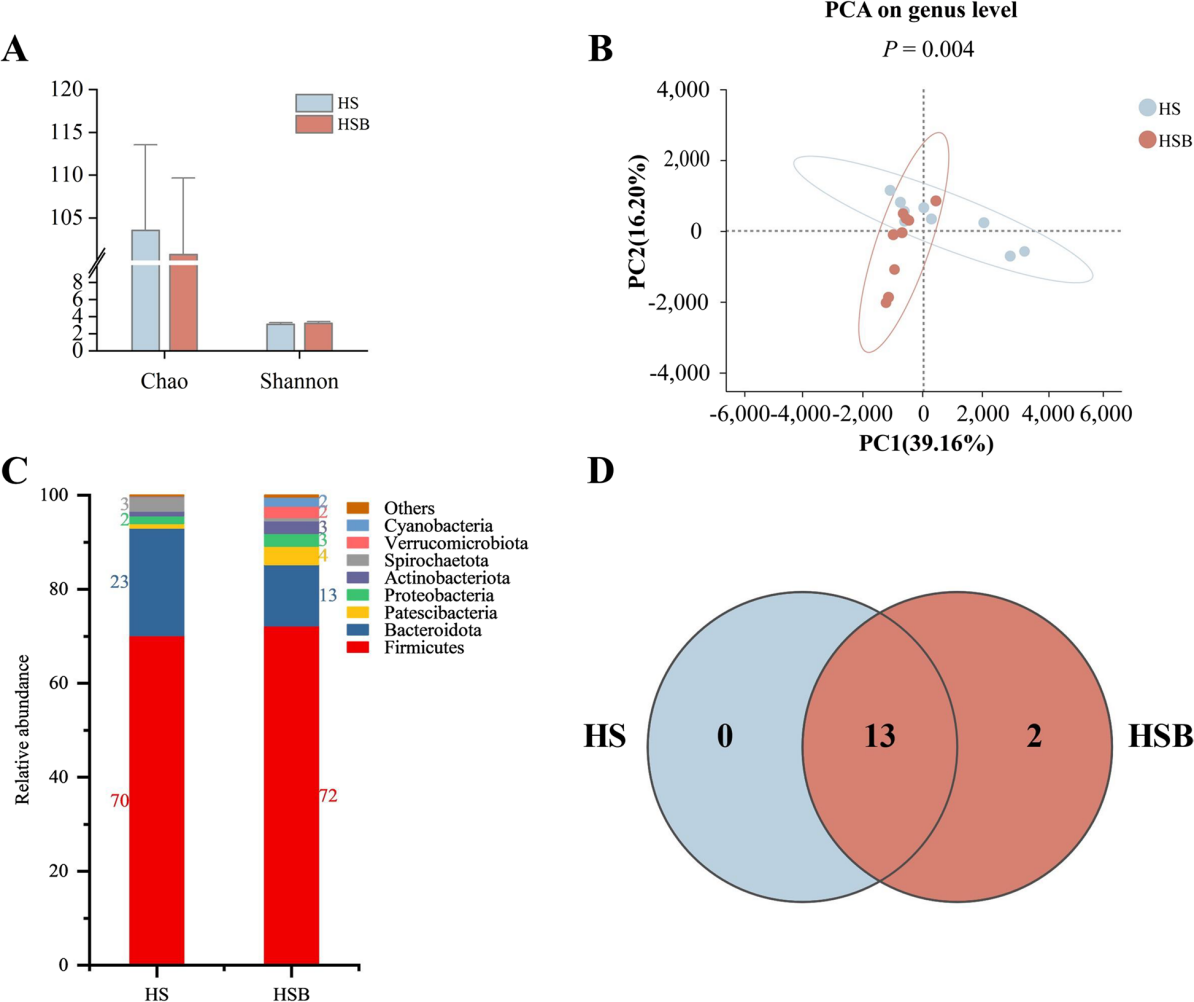
The differences in rectal microbiota diversity between HS and HSB groups (*n* = 9). **A** The Chao1 and Shannon indexes of rectum microbiota. **B** The principal component analysis (PCA) statistical significance was determined using analysis of similarities (Adonis) with 999 permutations at the genus level. **C** Relative abundances of rectal bacterial phylum in the HS and HSB group. **D** The Venn diagram indicates the numbers of rectal bacterial phylum level shared among the two groups. HS means fed starch-rich diet. HSB means fed a starch-rich diet supply of 4 g/d bile acids for dairy goats every day. The differences in data (**A**) were tested by the Wilcon rank-sum test. The bars represent mean ± SEM. ^*^P < 0.05

No significant difference was observed in the richness and alpha diversity of the rectal microbiota between the two groups (Fig. 4A). However, the results of PCA analysis indicated a noticeable variation in the rectal bacterial community composition between the two groups (*P* < 0.01, Fig. 4B).

**Fig. 5:**
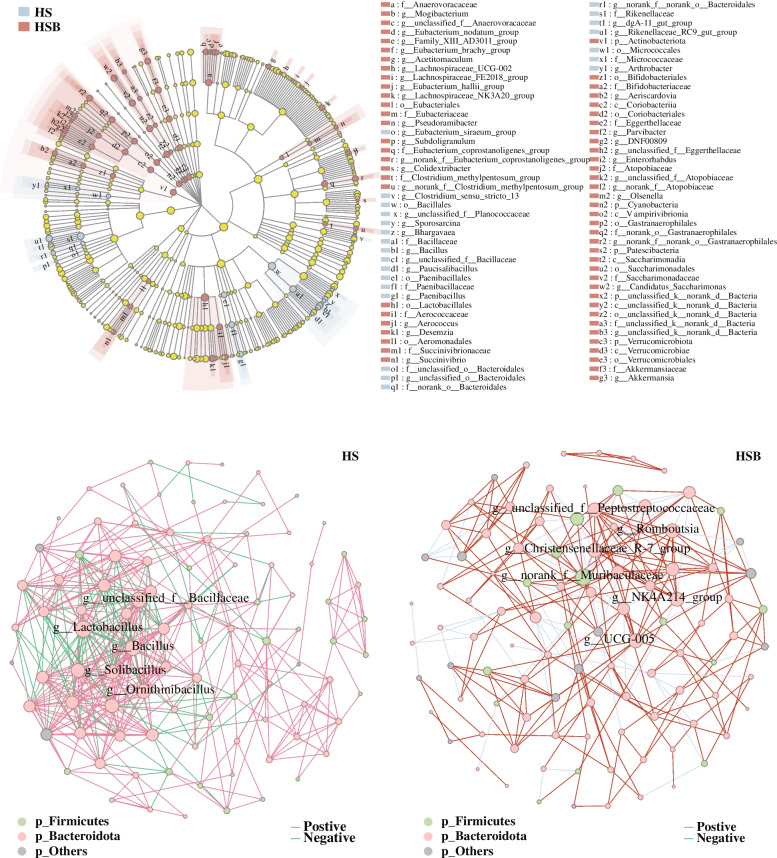
The differences in rectal microbiota structure and differentially abundant between HS and HSB groups (*n* = 9). **A** Linear discriminant analysis Effect Size (LEfSe) analysis between HS and HAB group. Co-occurrence network and network roles of the top 100 ASVs in the HS (**B**) and HSB (**C**) groups. Nodes represent ASVs, and the color of connecting lines indicates positive or negative correlations (Spearman, *P* < 0.05). No network hubs were identified in networks from both groups. HS means fed a starch-rich diet. HSB means fed a starch-rich diet supply 4 g/d bile acids for dairy goats every day

The analysis of bacterial family composition revealed that Firmicutes and Bacteroidetes were the predominant bacterial families in the rectal microbiota of goats (Fig. 4C), and the HSB group has two unique Deferribacterota and Planctomycetota at the phylum level (Fig. 4D). LEfSe analysis revealed significant enrichment in the HSB group, with a higher relative abundance of bacteria from phylum to genus levels. Analyses of the microbiota at the phylum level revealed a significant increase in the relative abundance of unclassified_k__norank_d__Bacteria*,* Patescibacteria, Actinobacteriota, Cyanobacteria, and Verrucomicrobiota. At the genus level, the result revealed that the HSB group highly enriched relative abundances of *Candidatus Saccharimonas*, *norank Eubacterium coprostanoligenes group*, *Akkermansia*, *Lachnospiraceae NK3A20 group*, *Family_XIII_AD3011_group*, *norank norank Gastranaerophilales*, and *Subdoligranulum*. On the other hand, the relative abundances of *Rikenellaceae RC9 gut group*, *Bacillus*, and *Paenibacillus* were lesser enriched in the HSB group (LDA > 3, *P* < 0.05, Fig. 5A). Co-occurrence network and network roles of the top 100 ASVs in the HS and HSB groups based on spearman correlation (|*r*| > 0.6, *P* < 0.05) analysis. BAs supplementation reduces the complexity of the microbial community. In the rectal microbial network of the HS group, the core taxa include *Bacillus*, *Lactobacillus*, *Solibacillus*, *Ornithinibacillus*, and *unclassified Bacillaceae* (Fig. 5B). On the other hand, the core taxa in the HSB group are *Christensenellaceae R-7 group*, *NK4A214 group*, *norank Muribaculaceae*, *Romboutsia*, and *unclassified Peptostreptococcaceae* (Fig. 5C).

### Bacterial abundance is correlated with BA composition in hindgut content

**Fig. 6:**
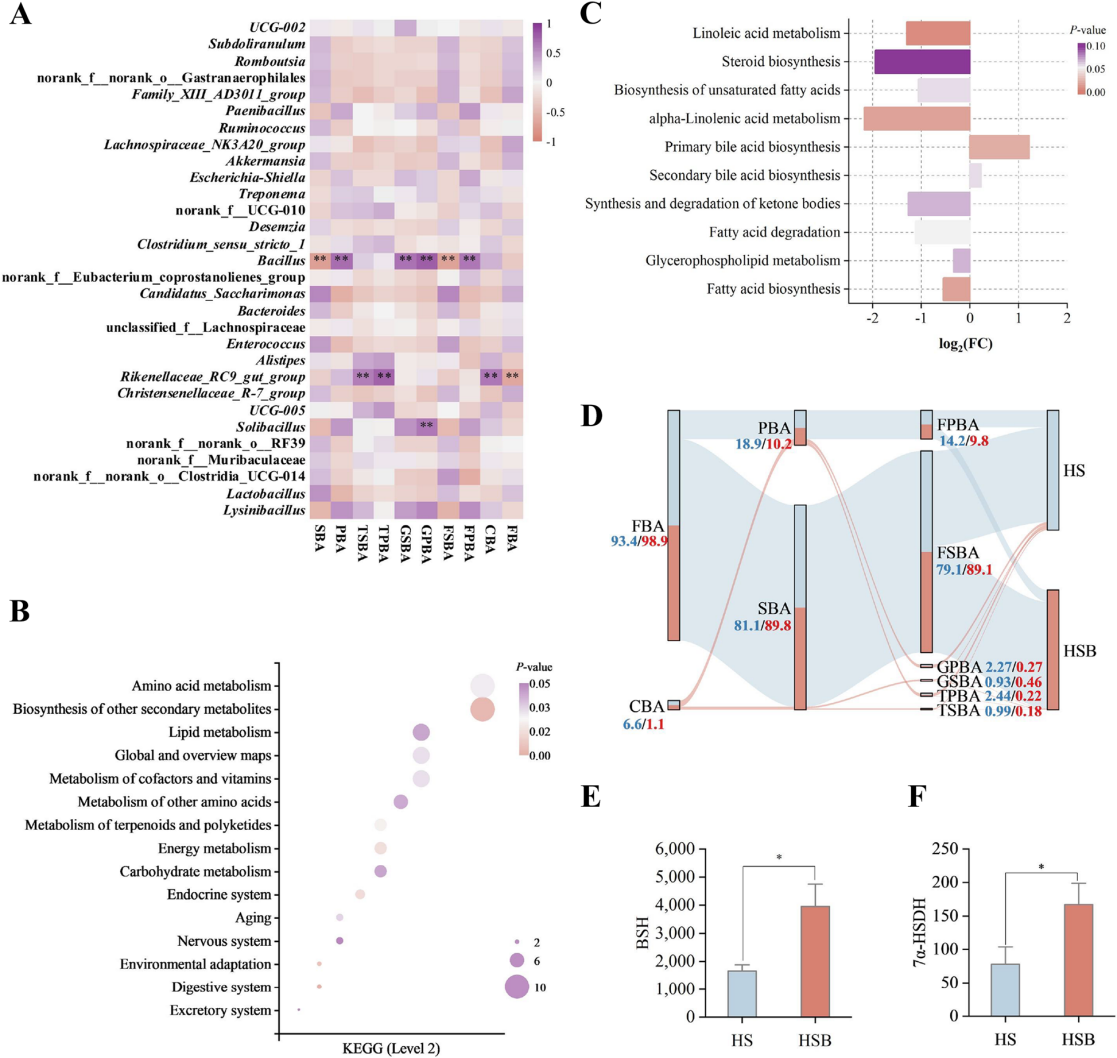
Lipid metabolism pathways in the KEGG pathway and the rectal microbiome-bile acids relationship in the HS and HSB group. (*n* = 9). **A** Correlations between the hindgut bile acids classification with rectal microbiomes. **B** Significantly different KEGG pathways (Level 2) of gut microbiome between HS and HSB groups.** C** KEGG pathways (Level 3) with lipid metabolism of gut microbiome between HS and HSB groups. **D** The flow among the different classifications of bile acids. **E** Based on PICRUSt2 functional prediction. Gene copy numbers of 7α-hydroxysteroid dehydrogenase (7α-HSDH) and **F** Bile acid hydrolase (BSH) in HS and HSB group. HS means fed starch-rich diet, HSB means fed starch-rich diet supply 4 g/d bile acids for dairy goats every day. The differences in data (**E**) were tested by the Wilcon rank-sum test. The bars represent mean ± SEM. ^*^P < 0.05. *PBA* Primary bile acid, *SBA* Secondary bile acid, *FBA* Free bile acid, *CBA* Conjugated bile acid, *FPBA* Free primary bile acid, *FSBA* Free secondary bile acid, *CPBA* Conjugated primary bile acid, *CSBA* Conjugated secondary bile acid, *GPBA* Glycine primary bile acid, *GSBA* Glycine secondary bile acid, *TPBA* Tauro-primary bile acid, *TSBA* Tauro-secondary bile acid

The hindgut content BA proportion were investigated using a spearman correlation (|*r*| > 0.6, *P* < 0.05) analysis to understand the relationship between hindgut content BA proportion and rectum bacterial at genus level (TOP30). The HS enriched bacteria *Rikenellaceae RC9 gut group* was positively correlated with the relative proportion of conjugated bile acid (CBA), tauro-PBA (TPBA) and tauro-SBA (TSBA) while negatively correlated with the relative proportion of FBA. *Bacillus* was positively correlated with the proportion of PBA, free primary bile acid (FPBA), GPBA and glyco-SBA (GSBA) while negatively correlated with the relative proportion of FSBA and SBA (Fig. 6B). The high BA proportion of HSB group was positively correlated with *norank Eubacterium coprostanoligenes group*, *Lachnospiraceae NK3A20 group*, and *Subdoligranulum*. Moreover, the unique BAs (6,7-DKLCA, GHCA, GHDCA, GUDCA) of HSB group were positively correlated with *Christensenellaceae R-7 group* (Fig. S3, |*r*| > 0.6, *P* < 0.05).

Functional prediction using PICRUSt2 showed significant differences (*P* < 0.05) in the functions related to lipid metabolism biosynthesis between the two groups. Among these KEGG pathways related to lipid metabolism, the abundance of 7 pathways was found to be significantly altered (*P* < 0.05) by BAs supplementation (Fig. 6B). Based on the KEGG pathways related to lipid metabolism, it was observed that their abundance decreased, except for PBA biosynthesis and SBA biosynthesis (Fig. 6C,  *P* < 0.05). In the hindgut BA pools, either FBA or SBA is present in the dominant position. Based on the different classifications of BAs, it was observed that the hindgut BA pool consisted of more than 80% FBA or SBA. However, in the HSB group, there was a higher proportion of FBA compared to the HS group (Fig. 6D). Additionally, the abundance of bacteria carrying the 7α-hydroxysteroid dehydrogenase (*7α-HSDH*) gene (*P* = 0.04), and bile salt hydrolase (*BSH*) gene (*P* < 0.01) were significantly higher in the HSB group (Fig. 6E and F).

## Discussion

The effects of BAs supplementation on various aspects of dairy goat physiology and metabolism were investigated in this study. The results revealed several interesting findings with implications for milk production, milk FA composition, rectal microbiota, and BA composition.

BAs supplementation positively impacted milk yield, suggesting its potential as a beneficial dietary supplement for enhancing milk production. Analysis of milk FA composition revealed that BAs supplementation promoted the accumulation of desirable preformed FAs in goat milk, potentially improving milk quality and nutritional value. BAs play a crucial role in lipid emulsification, facilitating lipid absorption through micelle formation in the intestine [9]. In particular, we observed a decrease in the proportion of C16:0 and an increase in the proportion of *cis*-9 C18:1 in the BAs supplementation group. These changes are noteworthy as C16:0 is a saturated fatty acid associated with potential health risks, while *cis*-9 C18:1 is a monounsaturated fatty acid known for its health benefits [27]. The decrease in C16:0 and the increase in *cis*-9 C18:1 proportion suggest that BAs supplementation may contribute to a healthier FA profile in goat milk. We observed a significant increase in the production of plasma TBA, suggesting an enhanced BA metabolism. The positive correlation between TBA and the proportion of preformed FAs, as well as the negative correlation with the proportion of mixed FAs, further supports the potential role of BAs in influencing milk FA composition. We obtained the plasma concentration of TBA in experiment, with a significant increase in the concentration of TBA in plasma after BAs supplementation. The increased concentration of TBA in the blood and hindgut contents sample before morning feeding suggested that BAs could reach the intestine to complete enterohepatic circulation and be transported through the systemic circulation to various parts of the body after supply. The improvement of BAs on lactation performance could be more pronounced when the diet contains more starch, as observed in previous studies, higher rumen degradable starch affects bile acid metabolism of dairy goats [20]. Previous study showed the concentrations of BAs (TLCA, TCA, and GCA) in plasma vary depending on the diet composition, with diets rich in whole grains generally exhibiting higher BA concentrations compared to refined grain diet [28]. Higher corn starch compounds may alter gut microbiota, which is involved in BA metabolism [29]. We used a starch-rich diet, which may have contributed to the observed improvement in lactation performance with BAs supplementation. Our results suggested that dietary supplementation with BAs could improve lactation performance in dairy goats under a starch-rich diet.

Furthermore, the analysis of BA composition in hindgut content revealed significant differences between the BAs supplementation group and the control group. The BA profile in the BAs supplementation group was characterized by higher proportions of certain BAs, such as HDCA, 3β-HDCA, and 6-ketoLCA. Previous studies have consistently demonstrated that the addition of BAs has a significant impact on the composition of the organism’s BA pool. When BAs are supplemented, changes in the relative proportions and concentrations of different BA species can be observed [30]. These differences in BA composition indicate that BAs supplementation influenced the enterohepatic circulation and metabolism of BAs in dairy goats. While fecal BA pools may not completely reflect the composition of intestinal BA pools, they do correlate with intestinal BA pools [31]. The group receiving BAs supplementation showed a higher proportion of FBA and a lower proportion of CBA in their BA composition. The conversion of CBA to FBA requires microbial involvement [32]. It also predicts higher levels of CBA are indeed predicted in the foregut. CBA, being more acidic, exhibit a superior lipid emulsification effect compared to their free form. Due to their acidic nature, CBA can exist as ions in the pH environment of the intestine, enhancing their emulsification capabilities [33]. This stronger emulsification effect aids in the breakdown of dietary fats into smaller droplets, facilitating their digestion and absorption in the small intestine. Therefore, the higher concentration of CBA in the foregut contributes to more efficient lipid digestion and absorption in the gastrointestinal tract.

In terms of rumen fermentation parameters, BAs supplementation did not significantly affect the ratio of SCFAs, indicated that the rumen fermentation was not significantly altered by BAs supplementation. Regarding the rectal microbiota, the PCA analysis revealed distinct variations in the bacterial community composition between the BAs supplementation group and the control group. The abundances of certain bacterial families, such as Patescibacteria, Succinivibrio, Actinobacteriota, Verrucomicrobiota, and Cyanobacteria, were higher in the BAs supplementation group. On the other hand, Rikenellaceae and Bacillaceae showed higher abundances in the control group. These findings suggest that BAs supplementation may selectively modulate the composition of the rectal microbiota, favoring the enrichment of specific bacterial taxa. The interaction between BAs and gut flora is well-established [34, 35], and BAs supplementation has been shown to have a profound impact on the microbial composition of the gut in various organisms, including monogastric animals [36] and aquatic species [37]. BAs play a crucial role in shaping the gut microbial community [38]. They have antimicrobial properties that help maintain a healthy balance of microorganisms in the gut [39]. Additionally, BAs can act as signaling molecules that influence the growth and activity of certain bacteria, thereby influencing the overall microbial composition [15, 40]. In our study, we investigated the effects of BAs supplementation on the gut microbiota in the dairy goats. We found that the BAs supplementation resulted in significant alterations in the microbial composition of the gut. Specific bacterial taxa were either enriched or depleted in response to BAs supplementation, indicating a direct influence on the gut microbiota. These findings highlight the importance of considering the interplay between BAs and gut flora when studying the effects of BAs supplementation. The microbial changes observed could have implications for various aspects of organismal health and metabolism. Further research is needed to elucidate the precise mechanisms through which BAs modulate the gut microbiota and to explore the functional consequences of these microbial alterations.

The correlation analysis between bacterial abundance and rectal BA composition provided additional insights into the relationship between the microbiota and BA metabolism. For instance, the enrichment of certain bacterial groups, such as *Rikenellaceae RC9 gut group* and *Bacillus*, was found to be positively correlated with specific types of BAs, such as PBA and FSBA. This suggests that these bacterial groups may play a role in the metabolism and transformation of BAs in the gastrointestinal tract. Additionally, the higher relative proportion of FBA, SBA, and FSBA in the BAs supplementation group further support the involvement of gut microbiota in BA metabolism. This suggests that these bacterial groups may play a role in the metabolism and transformation of BAs in the gastrointestinal tract. Additionally, the higher relative proportions of FBA, SBA, and FSBA in the BAs supplementation group further support the involvement of gut microbiota in BA metabolism. BAs (HCA, HDCA, and CDCA) supplementation has been demonstrated to impact glycolipid metabolism, partly through the modulation of signaling mechanisms such as TGR5 (Takeda G protein-coupled receptor 5) and FXR (farnesoid X receptor) [41–43]. These signaling pathways play crucial roles in regulating glucose and lipid metabolism, including the synthesis, storage, and utilization of carbohydrates and lipids [44–46]. Thus, BAs supplementation can potentially modulate glycolipid metabolism and contribute to metabolic regulation in the context of dietary interventions strategies. However, it is hypothesized that potential BAs may facilitate intestinal lipid absorption, thereby reaching the hindgut with diminished lipid content. Consequently, this process may result in the downregulation of pathways associated with lipid metabolism. The functional prediction analysis using PICRUSt2 revealed the abundance of several KEGG pathways related to lipid metabolism was significantly altered by BAs supplementation suggested that BAs supplementation may modulate lipid metabolism in dairy goats. Our findings are consistent with previous research that indicates a link between BAs and lipid mobilization in periparturient dairy cows [47]. This aligns with the observed alterations in KEGG pathways related to lipid metabolism in our study. Moreover, the changed in KEGG lipid functional pathways further support the impact of BAs on lipid metabolism. These pathways are involved in processes such as fatty acid synthesis, degradation, and transport, indicating a comprehensive modulation of lipid-related processes in response to BAs supplementation.

## Conclusions

In conclusion, our study provides novel insights into the application of BAs supplementation in dairy goats. Through our investigation, we observed significant shifts in the composition of the hindgut microbiota, coupled with alterations in BA composition. This interplay seemingly offers a possible plausible explanation for the observed improvements in the composition of preformed FAs resulting from BAs supplementation. Moreover, our findings suggest a potential interrelationship that could be influenced by the dynamic interplay among BAs, microbiota, and lipid metabolism when dietary BAs supplementation. This emerging understanding hints at possible connections between these factors and their potential influence on the milk quality of dairy goats.

Future research should focus on unraveling the underlying mechanisms of BAs’ effects on gut microbial ecology and exploring their potential health benefits. Investigating the long-term effects of BAs supplementation on animal health, milk-related products, and consumer preferences would provide valuable insights for practical applications in the dairy goats’ industry. Moreover, studying the interactions between BAs, gut microbiota, and host metabolism could shed light on the broader implications of BAs supplementation in animal nutrition and human health. Overall, our study opens up new avenues for research and theoretical support for utilizing BAs as a dietary strategy in dairy goats’ production systems.

### Supplementary Information

**Additional file 1: Table S1.** Ingredients and chemical composition of the experiment diet. **Table S2.** The effect of supplement bile acids on milk fatty acid composition of dairy goats fed a starch-rich diet (*n* = 9). **Table S3.** The effect of supplement bile acids on blood metabolites of dairy goats fed a starch-rich diet (*n* = 9) **Table S4.** The effect of supplement BAs on hindgut content BA composition of dairy goats fed a starch-rich diet (*n* = 9).

**Additional file 2: Fig. S1.** Correlations between the milk FA with blood metabolites (**A**) and rumen short chain fatty acid (**B**). TBA: Total bile acid; TG: Triglycerides; CHOL: Cholesterol; GLU: Glucose. De novo (FAs < 16 C) originates from de novo synthesis in the mammary gland, preformed (FAs > 16 C) originates from plasma, and mixed (FAs = 16 C) originate from both sources. SFA: Saturated fatty acid; MUFA: Monounsaturated fatty acid; PUFA: Polyunsaturated fatty acid. ^*^P < 0.05. **Fig. S2.** Significantly different relative abundance of bile acids classification in the hindgut of HS and HAB group. PBA: Primary bile acid; SBA: Secondary bile acid; FBA: Free bile acid; CBA: Conjugated bile acid; FPBA: Free primary bile acids; FSBA: Free secondary bile acid; CPBA: Conjugated primary bile acid; CSBA: Conjugated secondary bile acid; GPBA: Glyco-primary bile acid; GSBA: Glycine secondary bile acid; TPBA: Tauro-primary bile acid; TSBA: Tauro-secondary bile acid. **Fig. S3.** Correlations between the hindgut BA proportion with rectal microbiomes. 12-KLCA: 12-Ketolithocholic acid; 12-oxo-CDCA: 12-Oxochenodeoxycholic acid; 3-oxo-CA: 3-Oxocholic acid; 3-oxo-DCA: 3-Oxodeoxycholic acid; 3β-CA: 3β-Cholic acid; 3β-UDCA: 3β-Ursodeoxycholic acid; 6,7-DKLCA: 6,7-Diketolithocholic acid; 6-ketoLCA: 5-β-Cholanic acid-3α-ol-6-one; 7,12-DKLCA: 7,12-Diketolithocholic acid; 7-KDCA: 7-Ketodeoxycholic acid; 7-KLCA: 7-Ketolithocholic acid; CA: cholic acid; CA-3S: Cholic acid 3-sulfate sodium salt; CA-7S: Cholic acid 7-sulfate; CDCA: Chenodeoxycholic acid; CDCA-3Gln: Chenodeoxycholic acid-3-β-D-glucuronide; CDCA-3S: Chenodeoxycholic acid 3-sulfate disodium salt; DCA: Deoxycholic acid; DCA-3-O-S: Deoxycholic acid 3-O-sulfate disodium salt; DLCA: Dehydrolithocholic acid; GCA: Glycocholic acid; GCDCA: Glycochenodeoxycholic acid; GCDCA-3S: Glycochenodeoxycholic acid 3-sulfate disodium salt; GDCA: Glycodeoxycholic acid; GHCA: Glycohyocholic acid; GHDCA: Glycohyodeoxycholic acid; GLCA: Glycolithocholic acid; GUDCA: Glycoursodeoxycholic acid; GUDCA-3S: Glycoursodeoxycholic acid 3-sulfate sodium; HCA: Hyocholic acid; HDCA: Hyodeoxycholic acid; IALCA: Isoallolithocholic acid; ILCA: Isolithocholic acid; IsoCDCA: Isochenodeoxycholic acid; LCA: Lithocholic acid; LCA-3S: Lithocholic acid-3-sulfate; MDCA: Murideoxycholic acid; NCA: Norcholic acid; TCA: Taurocholic acid; TCA-3S: Taurocholic acid 3-sulfate sodium salt; TCDCA: Taurochenodeoxycholic acid; TDCA: Taurodeoxycholic acid; THDCA: Taurohyodeoxycholic acid; TLCA: taurolithocholic acid; TUDCA: Tauroursodeoxycholic acid; Tα-MCA: Tauro-α-muricholic acid; Tβ-MCA: Tauro-β-muricholic acid; UCA: Ursocholic acid; α-MCA: α-Muricholic acid; βGCA: 3β-Glycocholic acid; β-MCA: β-Muricholic acid; ω-MCA: ω-Muricholic acid.

## Data Availability

The authors confirm that all data underlying the findings are fully available without restriction.

## Abbreviations

BA: Bile acid
CA: Cholic acid
CBA: Conjugated bile acid
CDCA: Chenodeoxycholic acid
CHOL: Cholesterol
CSBA: Conjugated secondary bile acid
DCA: Deoxycholic acid
FA: Fatty acid
FPBA: Free primary bile acid
FSBA: Free secondary bile acid
GCA: Glycocholic acid
GHCA: Glycohyocholic acid
GHDCA: Glycohyodeoxycholic acid
GPBA: Glyco-primary bile acid
GUDCA: Glycohyodeoxycholic acid
HCA: Hyocholic acid
HDCA: Hyodeoxycholic acid
IALCA: Isoallolithocholic acid
isoCDCA: Isochenodeoxycholic acid
LDA: Linear discriminant analysis
LCA-3S: Lithocholic acid-3-sulfate
PBA: Primary bile acid
SBA: Secondary bile acid
SCFA: Short-chain fatty acid
TBA: Total bile acid
THDCA: Taurohyodeoxycholic acid
TPBA: Tauro-primary bile acid
TSBA: Tauro-secondary bile acid
TUDCA: Tauroursodeoxycholic acid
6-ketoLCA: 5β-Cholanic acid-3α-ol-6-one
6,7-DKLCA: 6,7-Diketolithocholic acid
βGCA: 3β-Glycocholic acid
ω-MCA: ω-Muricholic acid
