# Supplementary material for: Enhancing milk quality and modulating rectal microbiota of dairy goats in starch-rich diet: the role of bile acid supplementation
Source: J Anim Sci Biotechnol. 2024 Jan 22;15:7. doi: 10.1186/s40104-023-00957-7 (PMC10801996; doi:10.1186/s40104-023-00957-7)
Supplement: Supplementary file 1 — Additional file 1: Table S1. Ingredients and chemical composition of the experiment diet. Table S2. The effect of supplement bile acids on milk fatty acid composition of dairy goats fed a starch-rich diet (n = 9). Table S3. The effect of supplement bile acids on blood metabolites of dairy goats fed a starch-rich diet (n = 9) Table S4. The effect of supplement BAs on hindgut content BA composition of dairy goats fed a starch-rich diet (n = 9). [file 40104_2023_957_MOESM1_ESM.docx]

**Table S1** Ingredients and chemical composition of the experiment diet

| **Item^a^** | **Content** |
| --- | --- |
| Ingredient, % of DM |  |
| Alfalfa hay | 15.5 |
| Corn silage | 26.5 |
| Whole corn | 40.7 |
| Soybean meal | 13.6 |
| Limestone | 0.76 |
| Calcium hydrophosphate | 0.24 |
| Sodium bicarbonate | 0.78 |
| Salt | 0.48 |
| Fat powder | 1.00 |
| Vitamin-mineral mix^b^ | 0.48 |
| Chemical composition |  |
| DM, % | 60.4 |
| CP, % of DM | 16.6 |
| NDF, % of DM | 30.5 |
| ADF, % of DM | 19.6 |
| Starch, % of DM | 35.4 |

^a^ DM = dry matter, CP = crude protein, NDF = neutral detergent fiber, ADF = acid detergent fiber

^b^ Content per kilogram of product: Fe 1,310 mg, Cu 437 mg, Mn 833 mg, Zn 1,528mg, I 70 mg, Co 52 mg, Ca 35 mg, Se 26 mg, Vitamin A 381,000 IU, Vitamin D_3_ 39,900 IU, Vitamin E 1,450 IU

**Table S2** The effect of supplement bile acids on milk fatty acids composition of dairy goats fed a starch-rich diet (*n* = 9)

| **Item^a^, g/100g of total FA** | **Treatments^b^** | | **SEM^c^** | ***P*-value** |
| --- | --- | --- | --- | --- |
|  | **HS** | **HSB** |  |  |
| C4:0 | 0.56 | 0.52 | 0.052 | 0.49 |
| C6:0 | 1.09 | 0.97 | 0.079 | 0.16 |
| C8:0 | 1.95 | 1.69 | 0.142 | 0.08 |
| C10:0 | 9.15 | 7.90 | 0.483 | 0.02 |
| C11:0 | 0.15 | 0.17 | 0.033 | 0.45 |
| C12:0 | 6.39 | 5.67 | 0.498 | 0.17 |
| anteiso-C13:0 | 0.050 | 0.055 | 0.0179 | 0.68 |
| C13:0 | 0.15 | 0.15 | 0.022 | 1.00 |
| iso-C14:0 | 0.090 | 0.057 | 0.0171 | 0.11 |
| C14:0 | 12.8 | 12.6 | 0.85 | 0.85 |
| *cis*-9 C14:1 | 0.15 | 0.14 | 0.024 | 0.69 |
| anteiso-C15:0 | 0.31 | 0.26 | 0.061 | 0.37 |
| C14:1 | 0.33 | 0.33 | 0.057 | 0.96 |
| C15:0 | 0.93 | 1.04 | 0.131 | 0.43 |
| C15:1 | 0.032 | 0.041 | 0.0043 | 0.06 |
| iso-C16:0 | 0.17 | 0.15 | 0.048 | 0.69 |
| C16:0 | 31.4 | 28.7 | 1.06 | 0.02 |
| *trans*-9 C16:1 | 0.024 | 0.054 | 0.0150 | 0.07 |
| C16:1 | 0.89 | 0.94 | 0.142 | 0.73 |
| iso-C17:0 | 0.59 | 0.65 | 0.085 | 0.45 |
| anteiso-C17:0 | 0.31 | 0.28 | 0.037 | 0.53 |
| C17:0 | 0.44 | 0.52 | 0.046 | 0.10 |
| iso-C18:0 | 0.073 | 0.055 | 0.0125 | 0.18 |
| C17:1 | 0.27 | 0.37 | 0.044 | 0.05 |
| C18:0 | 7.84 | 7.71 | 0.905 | 0.89 |
| *trans*-9 C18:1 | 0.76 | 1.71 | 0.506 | 0.08 |
| *cis*-9 C18:1 | 18.9 | 21.8 | 1.11 | 0.02 |
| *cis*-11 C18:1 | 1.23 | 1.51 | 0.182 | 0.15 |
| *trans*-11 C18:1 | 0.089 | 0.158 | 0.039 | 0.09 |
| *trans*-9,12 C18:2 | 0.20 | 0.03 | 0.135 | 0.21 |
| *cis*-9,12 C18:2 | 1.81 | 2.30 | 0.384 | 0.22 |
| C20:0 | 0.12 | 0.14 | 0.020 | 0.45 |
| C18:3, n-6 | 0.12 | 0.14 | 0.021 | 0.52 |
| *cis*-9, *trans*-11 CLA | 0.34 | 0.72 | 0.191 | 0.07 |
| *trans*-10, *cis*-12 CLA | 0.053 | 0.054 | 0.0072 | 0.89 |
| C18:3, n-3 | 0.015 | 0.019 | 0.0016 | 0.05 |
| C20:2 | 0.020 | 0.019 | 0.0026 | 0.95 |
| C20:3, n-6 | 0.21 | 0.26 | 0.029 | 0.15 |
| C24:0 | 0.11 | 0.14 | 0.014 | 0.11 |

^a^ FA = fatty acids, CLA = conjugated linoleic acid; SFA = saturated fatty acids, MUFA = monounsaturated fatty acids, and PUFA = polyunsaturated fatty acids

^b^ HS means starch-rich diet, HSB means starch-rich diet supply 4 g/d bile acids for dairy goats every day

^c^ SEM = standard error of mean

**Table S3** The effect of supplement bile acids on blood metabolites of dairy goats fed a starch-rich diet (*n* = 9)

| **Item^a^** | **Treatments^b^** | | **SEM^c^** | ***P*-value** |
| --- | --- | --- | --- | --- |
|  | **HS** | **HSB** |  |  |
| TP, g/L | 75.5 | 72.5 | 1.61 | 0.20 |
| ALB, g/L | 29.6 | 28.2 | 0.52 | 0.09 |
| GLO, g/L | 45.9 | 44.3 | 1.47 | 0.49 |
| A/G | 0.66 | 0.63 | 0.026 | 0.55 |
| ALT, U/L | 27.4 | 21.9 | 1.54 | 0.10 |
| AST, U/L | 114 | 116 | 7.6 | 0.82 |
| AST/ALT | 4.41 | 5.45 | 0.404 | 0.07 |
| GGT, U/L | 63.3 | 58.0 | 3.0 | 0.21 |
| ALP, U/L | 102 | 77.0 | 7.0 | 0.07 |

^a^ TP = total protein, ALB =albumin, GLO = globulin, A/G = albumin/globulin, ALT = alanine aminotransferase, AST = aspartate aminotransferase, GGT = γ-glutamyl transferase, ALP = alkaline phosphatase, TBA = total bile acid, TG = triglycerides, CHOL = cholesterol, GLU = glucose

^b^ HS means starch-rich diet, HSB means starch-rich diet supply 4 g/d bile acids for dairy goats every day

^c^ SEM = standard error of mean

**Table S4** The effect of supplement BAs on hindgut content BA composition of dairy goats fed a starch-rich diet (*n* = 9)

| **Item^a^, g/100g of total BAs** | **Treatments^b^** | | **SEM^c^** | ***P*-value** |
| --- | --- | --- | --- | --- |
|  | **HS** | **HSB** |  |  |
| 12-KLCA | 6.0 | 3.0 | 0.61 | < 0.01 |
| 12-oxo-CDCA | 1.81 | 0.20 | 0.214 | 0.07 |
| 23-DCA | 0.122 | 0.017 | 0.0149 | 0.01 |
| 3-oxo-CA | 0.01444 | 0.00072 | 0.00341 | 0.07 |
| 3-oxo-DCA | 5.4 | 1.6 | 0.63 | < 0.01 |
| 3β-CA | 0.099 | 0.012 | 0.016 | 0.02 |
| 3β-DCA | 5.8 | 1.6 | 0.609 | 0.03 |
| 3β-HDCA | 0.039 | 18.800 | 2.1681 | < 0.01 |
| 6,7-DKLCA | - | 0.0046 | 0.00057 | < 0.01 |
| 6-ketoLCA | 0.18 | 18.13 | 2.115 | < 0.01 |
| 7,12-DKLCA | 0.532 | 0.064 | 0.1207 | 0.12 |
| 7-KDCA | 2.76 | 0.24 | 0.580 | 0.05 |
| 7-KLCA | 2.5 | 1.5 | 0.60 | 0.45 |
| CA | 10.49 | 0.85 | 1.799 | 0.01 |
| CA-3S | 0.569 | 0.033 | 0.116 | 0.03 |
| CDCA | 2.10 | 0.62 | 0.529 | 0.30 |
| CDCA-3Gln | 0.060 | 0.006 | 0.0099 | 0.01 |
| CDCA-3S | 0.168 | 0.012 | 0.0383 | 0.05 |
| DCA | 31.5 | 8.6 | 3.51 | < 0.01 |
| DCA-3-O-S | 0.0905 | 0.0074 | 0.01740 | 0.02 |
| DLCA | 1.2 | 0.2 | 0.35 | 0.16 |
| GCA | 2.43 | 0.25 | 0.507 | 0.04 |
| GCDCA-3S | 0.262 | 0.055 | 0.0494 | 0.05 |
| GDCA | 0.62 | 0.12 | 0.134 | 0.08 |
| GHCA | - | 0.0083 | 0.00187 | 0.02 |
| GHDCA | - | 0.12 | 0.0193 | < 0.01 |
| GLCA | 0.041 | 0.021 | 0.0246 | 0.48 |
| GUDCA | - | 0.13 | 0.022 | < 0.01 |
| GUDCA-3S | 0.1685 | 0.0089 | 0.0221 | 0.02 |
| HCA | 0.59 | 5.89 | 1.69 | 0.05 |
| HDCA | 1.5 | 24.1 | 2.98 | < 0.01 |
| IALCA | - | 0.068 | 0.0138 | 0.03 |
| ILCA | 0.35 | 0.54 | 0.092 | 0.38 |
| isoCDCA | 11.5 | 6.4 | 1.12 | 0.02 |
| LCA | 2.7 | 2.1 | 0.379 | 0.31 |
| LCA-3S | - | 0.00047 | 0.00302 | 0.31 |
| MDCA | 0.026 | 1.458 | 0.227 | < 0.01 |
| NCA | 0.132 | 0.013 | 0.0192 | 0.11 |
| TCA | 4.46 | 0.16 | 1.18 | 0.08 |
| TCA-3S | 1.264 | 0.055 | 0.259 | 0.03 |
| TCDCA | 0.236 | 0.044 | 0.0574 | 0.11 |
| TDCA | 0.526 | 0.038 | 0.0895 | 0.01 |
| THCA | 0.028 | 0.014 | 0.0061 | 0.35 |
| THDCA | 0.0057 | 0.0137 | 0.00222 | 0.02 |
| TLCA | 0.045 | 0.023 | 0.0093 | 0.19 |
| TUDCA | - | 0.033 | 0.0056 | < 0.01 |
| Tα-MCA | 0.1553 | 0.0064 | 0.0306 | 0.02 |
| Tβ-MCA | 0.0238 | 0.0012 | 0.00522 | 0.04 |
| Tω-MCA | 0.0455 | 0.0026 | 0.00951 | 0.03 |
| UCA | 0.107 | 0.031 | 0.0132 | < 0.01 |
| α-MCA | 0.268 | 0.091 | 0.0337 | 0.01 |
| βGCA | - | 0.0055 | 0.0765 | < 0.01 |
| β-MCA | 0.407 | 0.019 | 0.0014 | 0.01 |
| ω-MCA | 0.92 | 2.78 | 0.289 | < 0.01 |
