# Supplementary material for: Enhancing milk quality and modulating rectal microbiota of dairy goats in starch-rich diet: the role of bile acid supplementation
Source: J Anim Sci Biotechnol. 2024 Jan 22;15:7. doi: 10.1186/s40104-023-00957-7 (PMC10801996; doi:10.1186/s40104-023-00957-7)
Supplement: Supplementary file 2 — Additional file 2: Fig. S1. Correlations between the milk FA with blood metabolites (A) and rumen short chain fatty acid (B). TBA: Total bile acid; TG: Triglycerides; CHOL: Cholesterol; GLU: Glucose. De novo (FAs < 16 C) originates from de novo synthesis in the mammary gland, preformed (FAs > 16 C) originates from plasma, and mixed (FAs = 16 C) originate from both sources. SFA: Saturated fatty acid; MUFA: Monounsaturated fatty acid; PUFA: Polyunsaturated fatty acid. *P < 0.05. Fig. S2. Significantly different relative abundance of bile acids classification in the hindgut of HS and HAB group. PBA: Primary bile acid; SBA: Secondary bile acid; FBA: Free bile acid; CBA: Conjugated bile acid; FPBA: Free primary bile acids; FSBA: Free secondary bile acid; CPBA: Conjugated primary bile acid; CSBA: Conjugated secondary bile acid; GPBA: Glyco-primary bile acid; GSBA: Glycine secondary bile acid; TPBA: Tauro-primary bile acid; TSBA: Tauro-secondary bile acid. Fig. S3. Correlations between the hindgut BA proportion with rectal microbiomes. 12-KLCA: 12-Ketolithocholic acid; 12-oxo-CDCA: 12-Oxochenodeoxycholic acid; 3-oxo-CA: 3-Oxocholic acid; 3-oxo-DCA: 3-Oxodeoxycholic acid; 3β-CA: 3β-Cholic acid; 3β-UDCA: 3β-Ursodeoxycholic acid; 6,7-DKLCA: 6,7-Diketolithocholic acid; 6-ketoLCA: 5-β-Cholanic acid-3α-ol-6-one; 7,12-DKLCA: 7,12-Diketolithocholic acid; 7-KDCA: 7-Ketodeoxycholic acid; 7-KLCA: 7-Ketolithocholic acid; CA: cholic acid; CA-3S: Cholic acid 3-sulfate sodium salt; CA-7S: Cholic acid 7-sulfate; CDCA: Chenodeoxycholic acid; CDCA-3Gln: Chenodeoxycholic acid-3-β-D-glucuronide; CDCA-3S: Chenodeoxycholic acid 3-sulfate disodium salt; DCA: Deoxycholic acid; DCA-3-O-S: Deoxycholic acid 3-O-sulfate disodium salt; DLCA: Dehydrolithocholic acid; GCA: Glycocholic acid; GCDCA: Glycochenodeoxycholic acid; GCDCA-3S: Glycochenodeoxycholic acid 3-sulfate disodium salt; GDCA: Glycodeoxycholic acid; GHCA: Glycohyocholic acid; GHDCA: Glycohyodeoxycholic acid; GLCA: Glycolithocholic acid; G [file 40104_2023_957_MOESM2_ESM.docx]

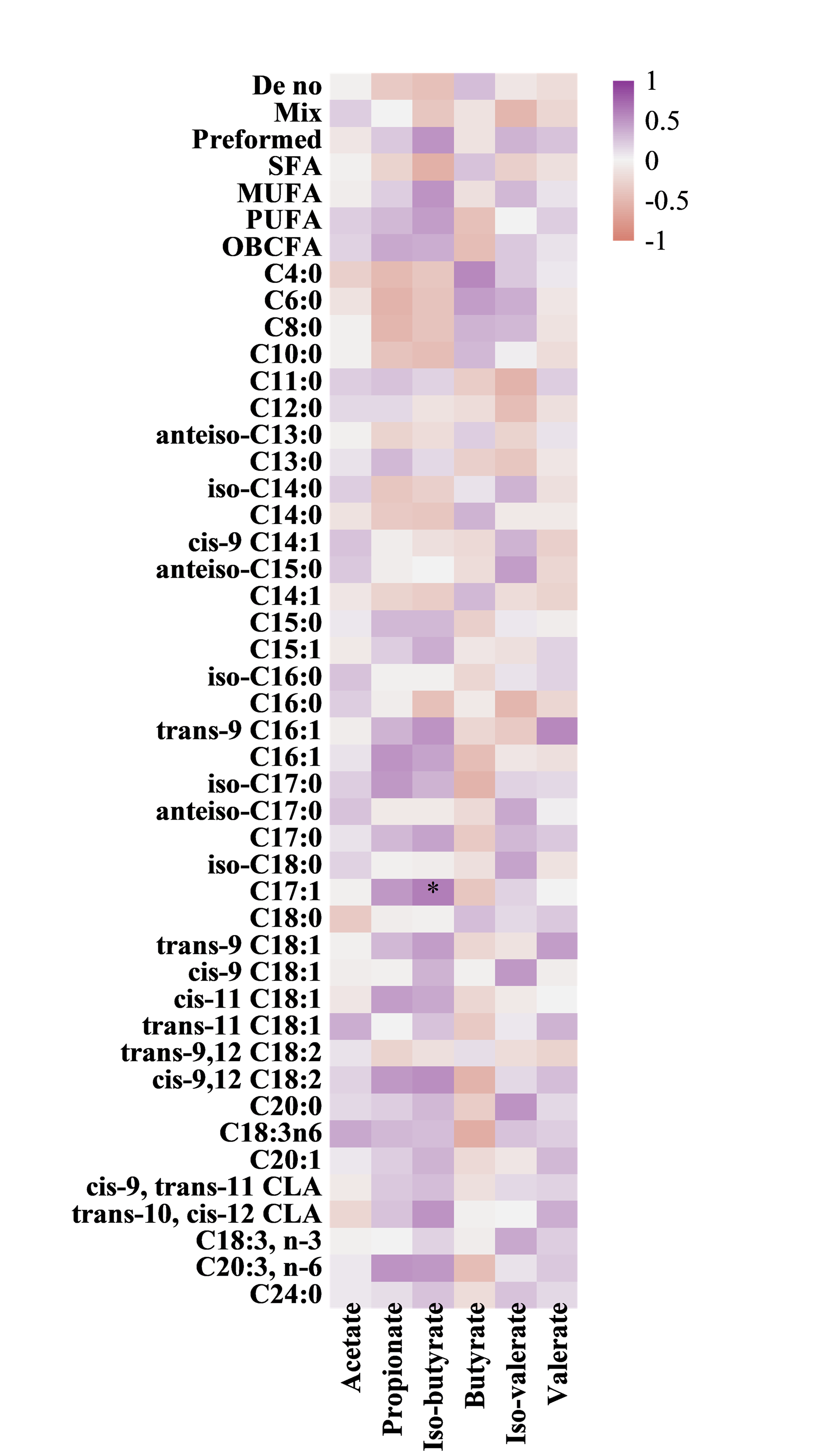

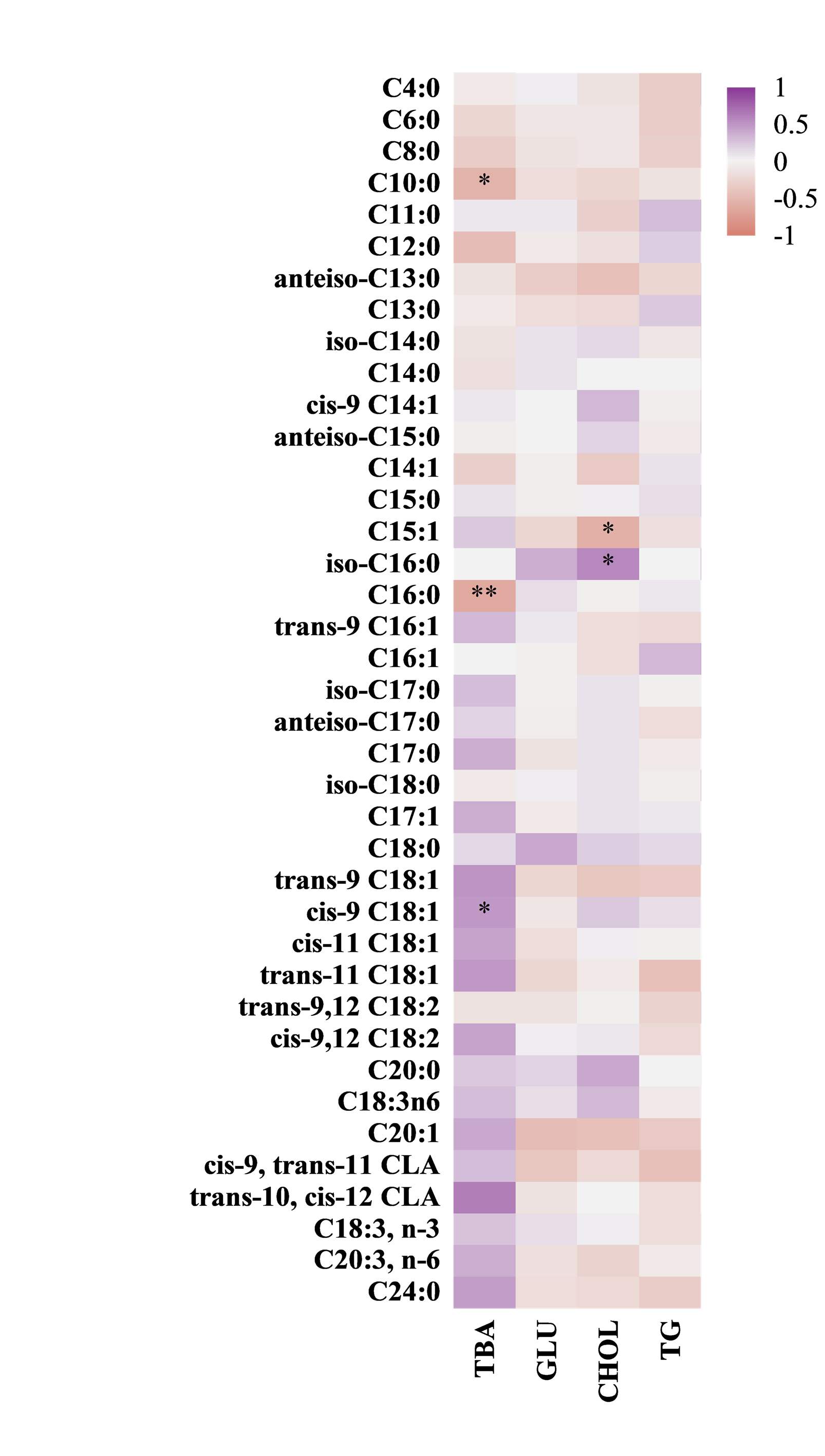
**Fig. S1** Correlations between the milk FA with blood metabolites (**A**) and rumen short chain fatty acid (**B**). TBA: Total bile acid; TG: Triglycerides; CHOL: Cholesterol; GLU: Glucose. De novo (FAs < 16 C) originates from de novo synthesis in the mammary gland, preformed (FAs > 16 C) originates from plasma, and mixed (FAs = 16 C) originate from both sources. SFA: Saturated fatty acid; MUFA: Monounsaturated fatty acid; PUFA: Polyunsaturated fatty acid. ^*^*P* < 0.05

**B**

**A**


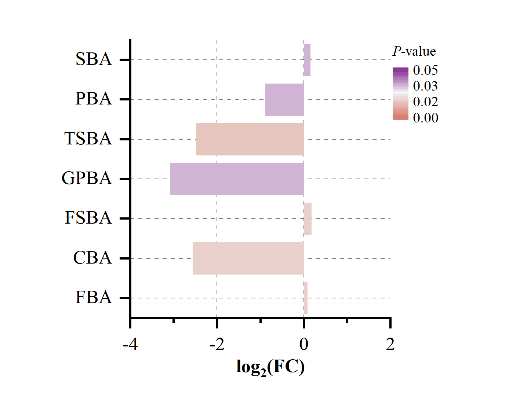


**Fig. S2** Significantly different relative abundance of bile acids classification in the hindgut of HS and HAB group. PBA: Primary bile acid; SBA: Secondary bile acid; FBA: Free bile acid; CBA: Conjugated bile acid; FPBA: Free primary bile acids; FSBA: Free secondary bile acid; CPBA: Conjugated primary bile acid; CSBA: Conjugated secondary bile acid; GPBA: Glyco-primary bile acid; GSBA: Glycine secondary bile acid; TPBA: Tauro-primary bile acid; TSBA: Tauro-secondary bile acid


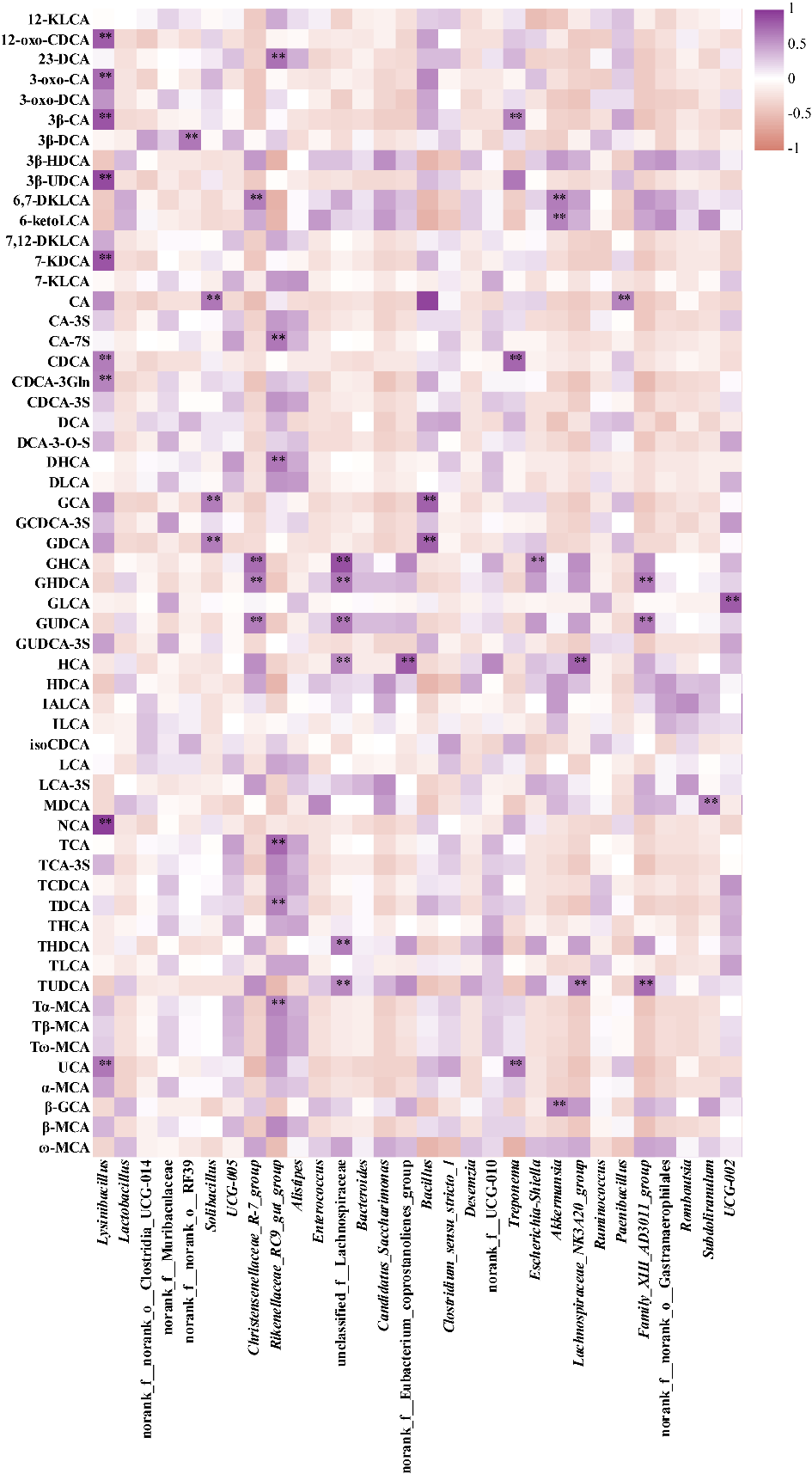


**Fig. S3** Correlations between the hindgut BA proportion with rectal microbiomes. 12-KLCA: 12-Ketolithocholic acid; 12-oxo-CDCA: 12-Oxochenodeoxycholic acid; 3-oxo-CA: 3-Oxocholic acid; 3-oxo-DCA: 3-Oxodeoxycholic acid; 3β-CA: 3β-Cholic acid; 3β-UDCA: 3β-Ursodeoxycholic acid; 6,7-DKLCA: 6,7-Diketolithocholic acid; 6-ketoLCA: 5-β-Cholanic acid-3α-ol-6-one; 7,12-DKLCA: 7,12-Diketolithocholic acid; 7-KDCA: 7-Ketodeoxycholic acid; 7-KLCA: 7-Ketolithocholic acid; CA: cholic acid; CA-3S: Cholic acid 3-sulfate sodium salt; CA-7S: Cholic acid 7-sulfate; CDCA: Chenodeoxycholic acid; CDCA-3Gln: Chenodeoxycholic acid-3-β-D-glucuronide; CDCA-3S: Chenodeoxycholic acid 3-sulfate disodium salt; DCA: Deoxycholic acid; DCA-3-O-S: Deoxycholic acid 3-O-sulfate disodium salt; DLCA: Dehydrolithocholic acid; GCA: Glycocholic acid; GCDCA: Glycochenodeoxycholic acid; GCDCA-3S: Glycochenodeoxycholic acid 3-sulfate disodium salt; GDCA: Glycodeoxycholic acid; GHCA: Glycohyocholic acid; GHDCA: Glycohyodeoxycholic acid; GLCA: Glycolithocholic acid; GUDCA: Glycoursodeoxycholic acid; GUDCA-3S: Glycoursodeoxycholic acid 3-sulfate sodium; HCA: Hyocholic acid; HDCA: Hyodeoxycholic acid; IALCA: Isoallolithocholic acid; ILCA: Isolithocholic acid; IsoCDCA: Isochenodeoxycholic acid; LCA: Lithocholic acid; LCA-3S: Lithocholic acid-3-sulfate; MDCA: Murideoxycholic acid; NCA: Norcholic acid; TCA: Taurocholic acid; TCA-3S: Taurocholic acid 3-sulfate sodium salt; TCDCA: Taurochenodeoxycholic acid; TDCA: Taurodeoxycholic acid; THDCA: Taurohyodeoxycholic acid; TLCA: taurolithocholic acid; TUDCA: Tauroursodeoxycholic acid; Tα-MCA: Tauro-α-muricholic acid; Tβ-MCA: Tauro-β-muricholic acid; UCA: Ursocholic acid; α-MCA: α-Muricholic acid; βGCA: 3β-Glycocholic acid; β-MCA: β-Muricholic acid; ω-MCA: ω-Muricholic acid
